# Cost-effectiveness of 7-day-Holter monitoring alone or in combination with transthoracic echocardiography in patients with cerebral ischemia

**DOI:** 10.1007/s00392-013-0601-2

**Published:** 2013-08-02

**Authors:** Felix Mayer, Raoul Stahrenberg, Klaus Gröschel, Sarah Mostardt, Janine Biermann, Frank Edelmann, Jan Liman, Jürgen Wasem, Alexander Goehler, Rolf Wachter, Anja Neumann

**Affiliations:** 1 Department of Cardiology and Pneumology, University of Göttingen, Göttingen, Germany; 2 Department of Neurology, University of Göttingen, Göttingen, Germany; 3 Institute for Health Care Management and Research, University of Duisburg-Essen, Campus Essen, Schützenbahn 70, 45127 Essen, Germany; 4 Department of Neurology, University of Mainz, Mainz, Germany; 5 Medical Informatics and Technology, University for Health Sciences, Hall, Austria; 6 Cardiac MR PET CT Program and Institute for Technology Assessment, Department of Radiology, Massachusetts General Hospital, Harvard Medical School, Boston, USA; 7 German Cardiovascular Research Center, Göttingen, Germany

**Keywords:** Atrial fibrillation, Stroke, Holter monitoring, Cost-effectiveness, Markov model

## Abstract

**Background and purpose:**

Prolonged Holter monitoring of patients with cerebral ischemia increases the detection rate of paroxysmal atrial fibrillation (PAF); this leads to improved antithrombotic regimens aimed at preventing recurrent ischemic strokes. The aim of this study was to compare a 7-day-Holter monitoring (7-d-Holter) alone or in combination with prior selection via transthoracic echocardiography (TTE) to a standard 24-h-Holter using a cost-utility analysis.

**Methods:**

Lifetime cost, quality-adjusted life years (QALY), and incremental cost-effectiveness ratios (ICER) were estimated for a cohort of patients with acute cerebral ischemia and no contraindication to oral anticoagulation. A Markov model was developed to simulate the long-term course and progression of cerebral ischemia considering the different diagnostic algorithms (24-h-Holter, 7-d-Holter, 7-d-Holter after preselection by TTE). Clinical data for these algorithms were derived from the prospective observational Find-AF study (ISRCTN 46104198).

**Results:**

Predicted lifelong discounted costs were 33,837 € for patients diagnosed by the 7-d-Holter and 33,852 € by the standard 24-h-Holter. Cumulated QALYs were 3.868 for the 7-d-Holter compared to 3.844 for the 24-h-Holter. The 7-d-Holter dominated the 24-h-Holter in the base-case scenario and remained cost-effective in extensive sensitivity analysis of key input parameter with a maximum of 8,354 €/QALY gained. Preselecting patients for the 7-d-Holter had no positive effect on the cost-effectiveness.

**Conclusions:**

A 7-d-Holter to detect PAF in patients with cerebral ischemia is cost-effective. It increases the detection which leads to improved antithrombotic regimens; therefore, it avoids recurrent strokes, saves future costs, and decreases quality of life impairment. Preselecting patients by TTE does not improve cost-effectiveness.

## Introduction

Stroke poses a heavy economic burden, accounting for ~2 to 7 % of total health expenditures that is equivalent to 0.15–0.36 % of the gross domestic product of western societies [1–3]. Atrial fibrillation (AF) is a frequent cause of ischemic stroke (IS) [4] and patients with AF have an almost fivefold increased risk of suffering a second stroke [5, 6]. Better detection of paroxysmal atrial fibrillation (PAF) by prolonged Holter monitoring [7] can be expected to improve secondary prevention through optimized secondary preventive regimens, namely, oral anticoagulation for those with PAF [8–10]. However, 7-day-Holter monitoring (7-d-Holter) is associated with increased costs. We therefore proposed to select patients with a high pre-test probability for PAF for extended Holter monitoring by applying a recently described index of left atrial enlargement and impaired left atrial function as measured by transthoracic echocardiography [11]. We aimed to evaluate the cost-effectiveness of three diagnostic strategies to detect PAF after acute cerebral ischemia: (a) standard 24-h-Holter monitoring (24-h-Holter), (b) 7-d-Holter, or (c) 7-d-Holter in a subgroup only, preselected by TTE (TTE/7-d-Holter).

## Methods

### Study population

The Markov model considers a hypothetical cohort of patients after the first ever IS or transient ischemic attack (TIA) presenting in sinus rhythm. In this cohort, AF has not previously been diagnosed and no contraindication of OAC therapy exists. The mean age is 68 years. Further details on the FIND-AF (ISRCTN 46104198) trial and its study population have been published elsewhere [7].

### Model structure and health states

**Fig. 1:**
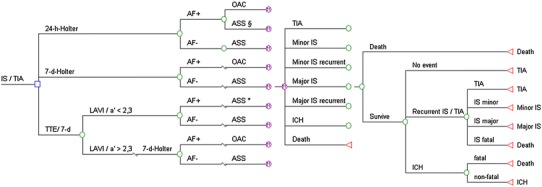
Decision model. It considers three diagnostic algorithms: 24-h-Holter monitoring referred to as standard diagnostic, 7-d-Holter and 7-d-Holter preceded by transthoracic echocardiography (TTE). *Section* displays false-negative results of 24-h-Holter and *asterisk* indicates false-negative results of TTE and, therefore, patients falsely treated with antiplatelets only. *AF+/AF−* patients with/without atrial fibrillation as a result of the three diagnostic algorithms, *ASS* aspirin, *ICH* intracranial hemorrhage, *IS* ischemic stroke, *LAVI* left atrial volume index, *OAC* oral anticoagulation, *TIA* transient ischemic attack, *TTE* transthoracic echocardiography

**Fig. 2:**
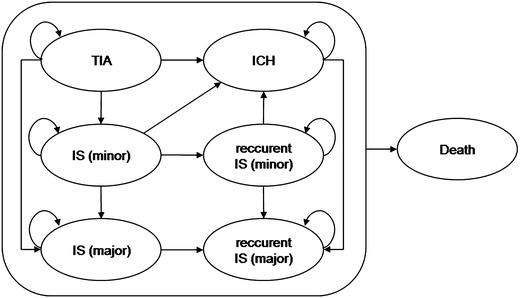
Possible transitions between permanent health states. Based on stroke severity within the FIND-AF cohort, patients start in one of the defined states namely TIA, minor stroke, major stroke, and recurrent minor or major stroke. Then patients cycle between states until death. The cycle length is 6 months. A transition to death from any cause (background mortality or fatal strokes) is possible from any state. *ICH* intracranial hemorrhage, *IS* ischemic stroke, *TIA* transient ischemic attack

We developed a Markov microsimulation model to estimate lifetime costs, cumulative quality-adjusted life years (QALYs), and the incremental cost-effectiveness ratio (ICER) of alternative diagnostic algorithms using TreeAge Pro Suite 2009 (TreeAge Software, Inc., Williamstown, Massachusetts). Markov modeling is a suitable technique in decision problems that involve risks and costs that can recur and/or change over time [12]. Our model analysis considered a lifelong horizon and we chose a cycle length of 6 months. The simulation was ended once the fraction of the cohort remaining alive fell below a threshold of 1 %. Costs of care were estimated from a third party payers’ perspective. Costs and quality of life estimates were discounted at a rate of 3 % [12]. After screening for PAF using 24-h-Holter, 7-d-Holter or TTE/7-d-Holter, patients entered a Markov model that simulated the long-term disease course and progression of cerebral ischemia, adverse events such as recurrent ISs, intracranial hemorrhage (ICH) or fatal strokes as well as dying from other causes. The risk of IS recurrence and ICH depended on the patient’s age and anticoagulation regimen. Our simulation model distinguished between seven permanent health states, namely TIA, minor stroke, major stroke, recurrent minor or major stroke, ICH and death. The general model structure and possible transitions between permanent health states are depicted in Figs. 1 and 2.

### Transthoracic echocardiography and detection rates of Holter monitoring

Our model compares three diagnostic algorithms/strategies to detect PAF. These strategies differed in detection rates of PAF, as derived from the FIND-AF cohort (Table 1). Prevalence of detected PAF was 13.3 % for 7-d-Holter [11]. Within our comparative approach, we considered 7-d-Holter to have 100 % sensitivity for PAF. The 24-h-Holter only detected PAF in 46.4 % of the patients who were positive using 7-d-Holter, leaving 53.6 % of the PAF cases undetected [7]. After preselecting patients for the 7-d-Holter using TTE (51.1 % of all patients had LAVI/a′ > 2.3 and therefore qualified for 7-d-Holter), the prevalence of PAF detected by 7-d-Holter was 23.9 %. Using the cut-off value of LAVI/a′ ≤ 2.3 as measured by TTE yielded 2.2 % of false negatives in FIND-AF [11]. The model considers patients receiving oral anticoagulation in form of warfarin or aspirin (ASS) as the most commonly used antiplatelet [13]. When PAF was detected by the 24-h-Holter or the 7-d-Holter, the treatment regimen was changed to warfarin, while all PAF-negative patients are treated with ASS (Fig. 1).

**Table 1:** Model variables: base case and range used in sensitivity analysis

Input variables	Base case	Range	References
Atrial fibrillation–detection parameters^a^ (%)			
Proportion of patients with LAVI/a′ ≤ 2.3	48.9	(44.01–53.79)	[11, 7], [FIND-AF]
Proportion of patients with LAVI/a′ > 2.3	51.1	(45.99–56.21)	[11, 7], [FIND-AF]
Negative predictive value (LAVI/a′ ≤ 2.3)	97.8	(88.02–100.0)	[11, 7], [FIND-AF]
False negatives (LAVI/a′ ≤ 2.3)	2.2	(1.98–2.42)	[11, 7], [FIND-AF]
Prevalence of AF (no preceding TTE)	13.3	(11.97–14.63)	[11, 7], [FIND-AF]
Prevalence of AF after preceding TTE	23.9	(21.51–26.29)	[11, 7], [FIND-AF]
Sensitivity of 24-h-Holter	46.4	(41.76–51.04)	[11, 7], [FIND-AF]
False negatives (24-h-Holter)	53.6	[48.24–58.96]	[11, 7], [FIND-AF]
Ischemic stroke parameters			
Annual rate of stroke with warfarin and AF^a,b^ (%)	3.02	(2.72–3.32)	[14, 15]
Ischemic strokes with warfarin that were			
Fatal (%)	8.2	(8.2–10.1)	[17]
Major (disabling) (%)	40.2	(40.2–41.7)	[17]
Minor (%)	42.5	(34.8–42.5)	[17]
TIA/no residua (%)	9.1	(9.1–13.3)	[17]
Relative risk of stroke with warfarin compared with aspirin	0.48	(0.37–0.63)	[15]
Annual rate of stroke with aspirin and AF^b^ (%)	6.3	(5.67–6.93)	[14]
Relative risk of stroke with AF compared to without AF	4.8	(2.0–6.0)	[6]
Annual rate of stroke with aspirin and without AF^b^ (%)	1.31	(1.18–1.44)	[6, 14]
Ischemic strokes with aspirin that were:			
Fatal (%)	17.9	(10.1–17.9)	[17]
Major (disabling) (%)	30.0	(30.0–41.7)	[17]
Minor (%)	41.0	(34.8–41.0)	[17]
TIA/no residua (%)	11.0	(11.0–13.3)	[17]
Hemorrhagic stroke parameters			
Annual rate of hemorrhagic stroke/ICH with warfarin^a,c^ (%)	1.28	(1.15–1.41)	[18]
Relative risk of hemorrhage with aspirin compared with warfarin	0.59	(0.5–0.7)	[15]
Annual rate of hemorrhagic stroke/ICH with aspirin^c^ (%)	0.76	(0.68–0.84)	[15, 18]
Mortality after hemorrhagic stroke/ICH	0.6	(0.46–0.68)	[20]
Mortality parameters, excluding acute stroke^a,d^			
Months 0–6 (%)	10.24	(9.22–11.26)	[21]
Months 6–12 (%)	6.20	(5.58–6.82)	[21]
Year 2–5 after stroke (%)	2.96	(2.66–3.26)	[21]
Year 6–15 after stroke (%)	6.76	(6.08–7.44)	[21]
Year 16+ after stroke (%)	9.15	(8.23–10.06)	[21]
Quality of life estimates^a^			
Ischemic stroke/major	0.52	(0.47–0.57)	[32]
Ischemic stroke/minor	0.87	(0.78–0.96)	[32]
Recurrent stroke (2nd disabling stroke)	0.12	(0.11–0.13)	[31]
TIA	0.9	(0.81–0.99)	[33]
Hemorrhagic stroke/intracranial hemorrhage (ICH)	0.62	(0.55–0.67)	[34]

*AF* atrial fibrillation, *ICH* intracranial hemorrhage, *LAVI* left atrial volume index, *TTE* transthoracic echocardiography *TIA* transient ischemic attack

^a^Range ±10 %

^b^Rate of stroke increased by the factor 1.4 per decade of life, compounded for every 6-month cycle

^c^Rate of hemorrhagic stroke/ICH increased by the factor 1.97 per decade of life, compounded for every 6-month cycle

^d^Mortality parameters adapted to a cycle length of 6 months

### Probability of adverse outcomes

To obtain key model inputs for IS/TIA recurrence, we reviewed relevant clinical trials and meta-analyses that investigated warfarin and aspirin therapy for secondary stroke prevention in patients with AF: 6.3 % annual rate of IS/TIA on ASS (false-negative detection) and a 0.48 relative risk of IS/TIA with warfarin compared to ASS resulting in 3.02 % annual rate on warfarin [14, 15]. Furthermore, we modeled a 4.8 relative risk of recurrent IS/TIA for patients with PAF compared to PAF-negative patients resulting in an annual recurrence rate of 1.31 % for PAF-negative patients treated with ASS [6, 14]. Annual rates for IS/TIA were increased by a factor of 1.4 per decade of life (multiplicative adjustment) to account for increasing age [16]. To account for different stroke severity levels, we classified IS into four categories: TIA, minor stroke, major stroke, and fatal stroke [17]. We considered an annual rate hemorrhagic stroke/intracranial hemorrhage of 1.28 % for warfarin and 0.76 % for aspirin (relative risk ASS vs. warfarin: 0.59) [15, 18]. Hemorrhage-related event rates were increased by a factor of 1.97 per decade of life (crude relative risk for every 10-year increase in age) [19]. Similarly to our consideration of different IS levels, we classified ICH into fatal and non-fatal events with a base-case mortality of OAC-associated ICH of 60 % [20]. Table 1 depicts all variables used in detail.

### Background mortality

Background mortality was modeled using age specific mortality rates adjusted for the increased risk of dying after cerebral ischemia [21]. These values reflect 6-month event rates after the initial event (Table 1) [22].

### Costs estimates

Since Find-AF was conducted in Germany, direct costs were estimated in a way such that it reflected the German health care system, i.e., DRG rate payments for hospitalization. All costs were adjusted to 2011 Euro using German consumer price indices.

### Acute care

Costs of acute care after recurrent events include those for emergency ambulance transportation, hospitalization, and inpatient (acute) rehabilitation (Table 2). Cost data which could not be extracted from the literature such as direct costs for acute hospitalization of patients with TIA were calculated using a nationwide base-rate of 2,936 € and a relative (cost) weight based on the appropriate DRG (B69) taken from the institute for the hospital remuneration system (InEK Begleitforschung) in Germany [23, 24]. The base rate was calculated by weighting the base rates of all German federal states (Verband der Ersatzkassen e. V.) by their total case mix as published by the InEK. Costs of acute hospitalization in patients with hemorrhagic strokes were calculated using a web DRG grouper based on the appropriate ICD as recommended by the German coding guidelines for neurological diseases (ICD I61, I69.1 und D68.30) and the nationwide base-rate [25, 26].

**Table 2:** Cost variables for acute treatment: base case and range used in sensitivity analysis

Input variables	Base case	Range	References
Cost of acute care^a,b^ (€)			
Ischemic stroke (with AF)	7,315	(6,584–8,047)	[27, 42]
Ischemic stroke (without AF)	6,224	(5,602–6,846)	[27, 42]
Ischemic stroke (fatal)	4,031	(3,628–4,434)	[43]
Hemorrhagic stroke	5,546	(4,991–6,100)	[23, 25–27]
Hemorrhagic stroke (fatal)	3,652	(3,287–4,017)	[43]
Transient ischemic attack (TIA)	2,637	(2,373–2,900)	[24, 27]
Additional resource cost 7-d vs. 24-h-Holter^a^ (€)	34	(20–165)	[FIND-AF], [29]
Cost discounting rate (%)	3	[1–5]	[12]

*AF* atrial fibrillation, *TIA* transient ischemic attack

^a^Presented in 2011 Euros

^b^Range ±10 %

### Post-acute care

For every subsequent 6-month cycle after cerebral ischemia we included costs for emergency ambulance transportation, hospitalization, outpatient care, medication, rehabilitation, reintegration, and nursing care (Table 3). Regarding post-acute costs of care, we distinguished between costs that occurred during the months 1–6, 7–12 and every following 6 months period after the initial event [27]. Calculation of medication costs was restrained to the different anticoagulation regimens under the assumption that medications for patients with PAF differ from those without PAF in the antithrombotic regimen only, by using the appropriate costs per defined daily dose (DDD) as published in the Arzneimittelverordnungsreport 2010 and adjusted these costs to reflect our cycle length [28]. INR measurements were not calculated separately, since they were included in the costs of outpatient care as reported by Brüggenjürgen et al. [27]. Post-acute costs varied by different health states and were considered every 6-month period after the initial event (Table 3). We assumed that rehabilitation, reintegration, and permanent nursing care were not necessary in patients who were not permanently compromised, and thus, did not include these costs in our analysis. Post-acute costs for TIA patients were limited to the costs of both anticoagulation regimens. Since there was limited data available considering post-acute costs of patients after ICH, we calculated these costs based on the relative difference in the utilities for patients after major IS and ICH (cost reduction 19.23 %).

**Table 3:** Cost variables: base case and range used in sensitivity analysis

Post-acute cost of care^a,b^ (€)	Month 1–6 after event		Month 7–12 after event		Every following 6-month period after year 1		References
	Base case	Range	Base case	Range	Base case	Range	
Ischemic stroke (major, aspirin)	7,224	(6,503–7,948)	6,420	(5,778–7,062)	5,635	(5,072–6,199)	[27, 28]
Ischemic stroke (major, warfarin)	7,265	(6,534–7,986)	6,460	(5,809–7,099)	5,674	(5,102–6,236)	[27, 28]
Ischemic stroke (minor, aspirin)	2,075	(1,868–2,283)	1,599	(1,439–1,759)	1,123	(1.011–1.235)	[27, 28]
Ischemic stroke (minor, warfarin)	2,115	(1,899–2,321)	1,639	(1,470–1,796)	1,163	(1,042–1,274)	[27, 28]
Hemorrhagic stroke (aspirin)	5,837	(5,252–6,420)	5,186	(4,667–5,704)	4,551	(4,096–5,006)	[27, 28]
Hemorrhagic stroke (warfarin)	5,871	(5,278–6,450)	5,220	(4,692–5,734)	4,585	(4,121–5,037)	[27, 28]
TIA (aspirin)	8	(7.2–8.8)	8	(7.2–8.8)	8	(7.2–8.8)	[28]
TIA (warfarin)	42	(37.8–46.2)	42	(37.8–46.2)	42	(37.8–46.2)	[28]

*TIA* transient ischemic attack

^a^Presented in 2011 Euros, differentiated by stroke severity and anticoagulation regimen

^b^Range ±10 %

### Resource costs of prolonged Holter monitoring

For the last 21 patients who had received the 7-d-Holter in Find-AF, detailed data on resource utilization were collected and compared to the 24-h-Holter. Based on these data, additional costs were added once for every patient in the 7-d-Holter strategy and those patients who received 7-d-Holter in the TTE/7-d-Holter strategy. To estimate costs for medical staff, costs per minute were calculated according to the gross annual salary and mean annual working time of medical practitioners and medical technical assistants in Germany [29, 30]. Preceding TTE was considered to be a standard procedure for patients with acute cerebral ischemia during hospitalization resulting in no additional costs (Table 2).

### Quality of life estimates

To calculate the quality-adjusted survival, literature search was done to obtain suitable quality of life estimates (utilities) for each health state modeled (Table 1) [17, 31]. For major stroke, the mean utility was 0.52 and 0.87 for minor stroke [32]. For recurrent disabling strokes—i.e., a second major stroke or a major stroke following ICH—we used a mean utility of 0.12 [31]. For patients after TIA we used a utility of 0.9 and for ICH 0.62 [33, 34]. The utility of death from any cause was 0. Patients remained in their original state when a recurrent event was less severe (e.g., TIA in patients with previous major stroke).

### Sensitivity analyses

We performed sensitivity analyses for all variables used in the model. Where no data about the plausible range of the input variable were available it was varied ±10 %.

## Results

### Base-case analysis

In the base-case scenario, the discounted quality-adjusted life expectancy in a 68-year-old patient with acute IS or TIA ranged from 3.833 QALYs for patients with 24-h-Holter to 3.842 QALYs with TTE/7-d-Holter and 3.844 QALYs with 7-d-Holter. Mean discounted lifetime costs ranged from 32,887 € for the TTE/7-d-Holter to 32,912 € for the 24-h-Holter (Table 4); 24-h-Holter was dominated by 7-d-Holter and TTE/7-d-Holter, respectively. The latter strategy had slightly lower mean lifetime costs, saving 8.9 € compared to the 7-d-Holter. In the base-case scenario this resulted in additional costs per QALY gained of 5,354 €/QALY for the 7-d-Holter compared to the TTE/7-d-Holter.

**Table 4:** Projected costs and QALYs for patients after ischemic stroke or TIA under base-case conditions and by varying risk of ischemic/hemorrhagic stroke

Rate of IS and ICH with warfarin (% per year)	ECG setting	Cost (€)	Incremental cost (€)	QALYs	Incremental effect (QALYs)	ICER (€ per QALY)
IS: 2.72 %, ICH: 1.15 %	TTE / 7-d-Holter	32,854.4	–	3.846	–	Reference
	7-d-Holter	32,860.5	6.1	3.848	0.0020	2978.37
	24-h-Holter	32,895.9	35.4	3.835	−0.0135	Dominated
IS: 3.02 %, ICH: 1.28 %	TTE / 7-d-Holter	32,886.9	–	3.842		Reference
Base case	7-d-Holter	32,895.8	8.9	3.844	0.0017	5,353.92
	24-h-Holter	32,912.3	16.5	3.833	−0.0111	Dominated
IS: 3.32 %, ICH: 1.41 %	TTE / 7-d-Holter	32,918.0	–	3.838	–	Reference
	24-h-Holter	32,928.0	10.0	3.831	−0.0073	Dominated
	7-d-Holter	32,929.7	11.7	3.839	0.0013	8,957.96
IS: 3.62 %, ICH: 1.54 %	24-h-Holter	32,943.2	–	3.829	–	Reference
	TTE/7-d-Holter	32,948.0	4.9	3.834	0.0053	913.25
	7-d-Holter	32,962.4	14.3	3.835	0.0009	15,145.35

*IS* ischemic stroke, *ICH* intracranial hemorrhage, *TTE* transthoracic echocardiography, *QALY* quality-adjusted life year, *ICER* incremental cost-effectiveness ratio

In a cohort of 10,000 patients with acute cerebral ischemia, the 7-d-Holter detected 710 more cases of PAF than the 24-h-Holter; this resulted in a gain of 7.85 QALYs, or 82 more cases than TTE/7-d-Holter, resulting in 0.14 QALYs gained. Over the patients’ lifetime, the 7-d-Holter prevented 155 IS compared to the 24-h-Holter but oral anticoagulation caused an additional 26 ICH (7-d-Holter vs. TTE/7-d-Holter: IS: −3; ICH: +5).

### Sensitivity analysis

**Fig. 3:**
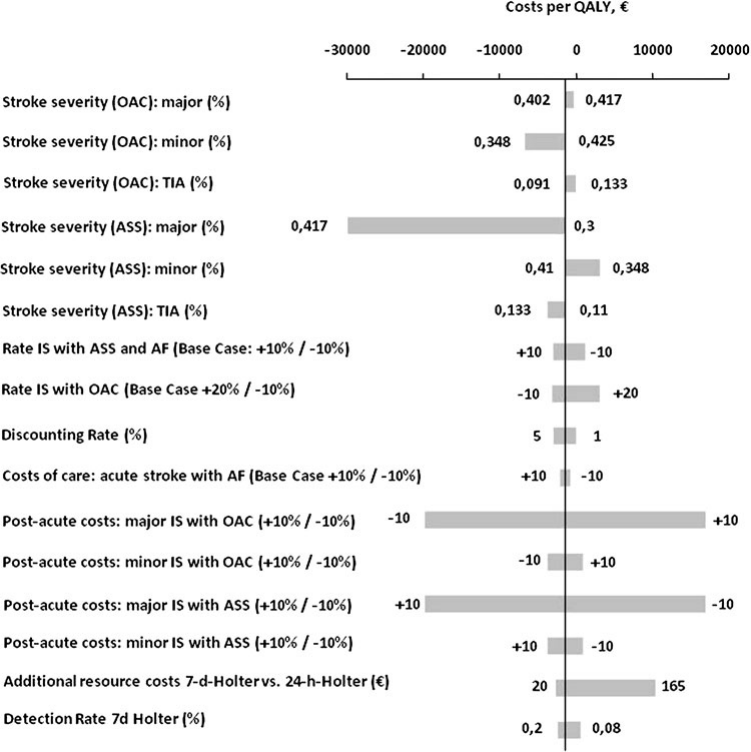
Univariate sensitivity analysis on most influential variables 7-d vs. 24-h-Holter monitoring: incremental cost-effectiveness ratio (ICER). Figure 3 displays the influence of a variation of variables used in the model on the ICER of the 7-d vs. 24-h-Holter. The vertical line represents the base-case scenario. Negative ICERs imply dominance of the 7-d-Holter and positive ICERs show the maximal costs per QALY gained. *AF* atrial fibrillation, *ASS* aspirin, *IS* ischemic stroke, *OAC* oral anticoagulation, *QALY* quality-adjusted life year, *TIA* transient ischemic attack

Examining the key model input parameters over a wide range demonstrated that the 7-d-Holter was cost-effective or dominant in most scenarios when compared to the 24-h-Holter (Fig. 3). A variation of stroke severity of recurrent IS of ASS- or OAC-treated patients influenced the incremental cost per QALY, but never exceeded 3,022 € per QALY gained. A 10 % increase in the annual risk of recurrent stroke in PAF-positive patients treated with ASS led to the dominance of the 7-d-Holter over the 24-h-Holter, while a 10 % reduction resulted in an ICER of 1,131 €/QALY.

Two-way sensitivity analysis investigating the annual recurrence of IS and ICH yielded an ICER of 15,145 €/QALY comparing the use of a 7-d-Holter to a 24-h-Holter when both event rates were increased by 20 %; a decrease of 10 % demonstrated the dominance of the 7-d-Holter (Table 4).

Variation in costs had the most influence on changes in the ICER; a 10 % increase in post-acute costs for patients after major IS and warfarin therapy estimated an ICER of 16,911 €/QALY, while a 10 % decrease in post-acute costs for similar patients treated with ASS yielded an ICER of 16,945 €/QALY. Extended use of Holter monitoring affected the ICER in two ways, a higher detection rate and extra costs due to the additional use of personnel and material resources. We varied the prevalence of PAF detected by 7-d-Holter from 8 to 20 %, yielding an ICER of 527 €/QALY at 8 %. Higher detection rates of up to 20 % enhanced the dominance of 7-d-Holter with a threshold of 8.9 % beyond which the 7-d-Holter was less expensive than the 24-h-Holter. Raising additional resource costs for 7-d-Holter to 165 € resulted in an ICER of 10,385 €/QALY. The 7-d-Holter remained dominant up to a threshold of 50.2 €.

With regard to test accuracy, 7-d-Holter remained cost-effective when compared to the TTE/7-d-Holter in most scenarios. Beyond a negative predictive value of 98.9 % for the preceding TTE, the TTE/7-d-Holter became the dominant strategy. An increase of PAF-prevalence without TTE up to 20 % resulted in dominance of 7-d-Holter; a decrease of PAF-prevalence with preceding TTE resulted in an ICER 7-d-Holter vs. TTE/7-d-Holter of 19,491 €/QALY at 21.5 %. Raising monitoring costs to 165 € increased the incremental cost-effectiveness ratio to 43,799 € per QALY gained because of the higher proportion of patients being examined by 7-d-Holter.

## Discussion

Our analysis demonstrated that using the 7-d-Holter instead of the standard 24-h-Holter to detect PAF in patients after cerebral ischemia is cost-effective. Given that prolonged Holter monitoring detects a higher number of new cases with PAF [35–37], the improved cost-effectiveness is attributable to the fact that these newly detected patients benefit from warfarin therapy to prevent stroke recurrence which in turns saves future costs.

Kamel and colleagues [38] previously examined the effect of an additional 7 days of outpatient cardiac monitoring vs. standard care to detect PAF after IS in the US. In this analysis, the detection rate of PAF was estimated to be 5.9 % using outpatient monitoring which resulted in an incremental cost-effectiveness ratio of 13,000 $/QALY, considerably below a threshold of 50,000 $ per QALY usually considered cost-effective [38, 39]. A more realistic scenario, for instance, a scenario with a higher detection rate of PAF by 7-d-Holter ECG improves the cost-effectiveness to 5,090 $/QALY gained [40], comparable to the data of our study. Instead of extrapolating trial data of non-continuous ECG recordings to estimate the yield of 7-d-Holter, we used actual observations from the Find-AF trial that offered intra-individual comparison of detection rates for 24-h, 48-h, and 7-d-Holter [7] in an unselected cohort of patients with cerebral ischemia. Furthermore, we prospectively assessed incremental costs for the performance of 7-d-Holter instead of 24-h-Holter in a subgroup of patients at the end of the FIND-AF trial, i.e., on top of the learning-curve for this method. In this respect, we believe that our analysis is based on solid and reliable data and our results are definitely supportive of the analysis by Kamel [38]. However in our base case, 7-d-Holter was not only cost-effective, but actually dominated the 24-h-Holter and remained highly cost-effective in the most unfavorable constellations on sensitivity analyses. In addition to supporting Kamel’s analysis with an alternative model based on clinical data, our analysis was performed for a different healthcare system (i.e., the German).

Our model predicts mean undiscounted lifetime costs for patients after the first ever IS or TIA of about 46,000 € and, therefore, slightly lower than those previously reported by Kolominsky-Rabas with 50,507 €. Compared to 7.3 years as reported by Kolominsky-Rabas, mean undiscounted life expectancy as predicted by our model ranged from 7.77 to 7.79 years [3]. These roughly similar figures give some external validation for our model.

To project IS recurrence, we used variables primarily based on values reported in the ACTIVE W trial [14]. To account for varying risks in PAF-positive and PAF-negative patients and to display the efficacy of warfarin and aspirin therapy within these cohorts, relative risks were used as reported in the literature [6, 15].

Regarding hemorrhagic complications of OAC treatment, we used the annual rate for ICH for patients with prior stroke or TIA taken from the RELY trial [18] and applied the relative risk ASS vs. OAC to project this event rate for ASS-treated patients [15, 18]. Other hemorrhagic complications such as abdominal or gastrointestinal bleeding were not included in our analysis. Furthermore, we assumed that a detection of PAF by Holter monitoring results in a change of treatment to OAC therapy in any case, although contraindications of oral anticoagulation may be present in some cases. These points therefore give some limitation to our analyses. However, given these limitations and the fact that newer anticoagulation drugs (NOACs) have just recently emerged to the market, which may even further reduce stroke recurrence in patients with AF with a similar or even lower risk for cerebral bleedings [41], prolonged Holter monitoring might became even more cost-effective.

Of note, the use of routine TTE to rule out PAF and to selectively apply the 7-d-Holter to a subgroup did not increase the cost-effectiveness ratio, due to the false-negative patients missed in this approach. Because these 2.2 % of patients would have been treated erroneously with antiplatelet therapy only, they would have had a higher risk of dying from recurrent events and, therefore, a reduction in cumulative quality-adjusted life expectancy. In sensitivity analyses, the TTE/7-d-Holter became the dominant strategy beyond a negative predictive value of 98.9 %. It should be noted that both false-negative patients in Find-AF only had one very short episode (about 40–45 s) of PAF on the 7-d-Holter. Because some might argue that PAF has only been shown to increase the risk of thromboembolism when episodes of at least 5–6 min have been documented, interpretation of our results would then have to be modified, as TTE would preselect with 100 % negative predictive value, making the TTE/7-d-Holter the dominant diagnostic strategy.

## Conclusions

The use of a 7-d-Holter as opposed to a standard 24-h-Holter in patients with cerebral ischemia is cost-effective across a wide range of variation of key variables and model input data.
